# Exploring frailty in Brazil: an analysis of the ELSI-Brazil survey

**DOI:** 10.1590/0102-311XEN041624

**Published:** 2025-04-28

**Authors:** Henrique Pott, Mario Ulises Pérez-Zepeda, Melissa K. Andrew, Kenneth Rockwood

**Affiliations:** 1 Universidade Federal de São Carlos, São Carlos, Brasil.; 2 Instituto Nacional de Geriatría, Ciudad de México, México.; 3 Dalhousie University, Halifax, Canada.

**Keywords:** Frailty, Aged, Frail Elderly, Health Care Surveys, Health Surveys, Fragilidade, Idoso, Idoso Fragilizado, Pesquisas sobre Atenção à Saúde, Inquéritos Epidemiológicos, Fragilidad, Anciano, Anciano Frágil, Encuestas de Atención de la Salud, Encuestas Epidemiológicas

## Abstract

The Brazilian aging population will challenge publicly funded health services, on which most Brazilians rely. The country must prepare for aging-associated health challenges such as frailty. We used data from the *Brazilian Longitudinal Study of Aging* (ELSI-Brazil) to generate a standardized Frailty Index (FI), assess frailty levels among this population, and supply reliable and nationwide information. In total, 9,901 adults aged 50 years or older were studied in the second wave of ELSI-Brazil. A 53-item FI was created according to a standardized protocol. Logistic regression was used to determine the association between frailty levels and disability/health status, whereas the relationship between frailty level, disabilities, and healthcare use was analyzed by a negative binomial regression. Frailty was high, with a 0.19 weighted mean FI score and 0.19 median. Frailty distribution was right-skewed, with higher levels in women and increased exponentially with age. Widow(er)s, black and mixed-race individuals, and those living in rural areas had higher levels of frailty. Regression models showed that higher frailty was associated with poorer self-assessment of health, higher disability, and greater use of healthcare services. This study shows a high prevalence of frailty in Brazilian middle-aged and older adults and its association with disability, health status, and healthcare service use. These relevant findings can inform healthcare policies and design services prioritizing this population’s health, particularly for those using public healthcare.

## Resumo

A população idosa do Brasil representará um desafio para os serviços públicos de saúde, dos quais a maioria dos brasileiros depende. O país deve se preparar para os desafios de saúde relacionados ao envelhecimento, como a fragilidade. Dados do *Estudo Longitudinal da Saúde dos Idosos Brasileiros* (ELSI-Brasil) foram empregados para gerar um Índice de Fragilidade (IF) padronizado, avaliar os níveis de fragilidade nessa população e fornecer informações confiáveis e de abrangência nacional. Na segunda onda do ELSI-Brasil, 9.901 adultos com 50 anos ou mais foram estudados. Seguindo um protocolo padronizado, um IF de 53 itens foi construído. A regressão logística foi usada para determinar a associação entre o nível de fragilidade e a incapacidade/estado de saúde, enquanto a regressão binomial negativa analisou a relação entre o nível de fragilidade, as incapacidades e o uso dos serviços de saúde. A fragilidade foi elevada, com uma pontuação média ponderada do IF de 0,19 e mediana de 0,19. A distribuição da fragilidade apresentou assimetria positiva, com níveis mais elevados em mulheres e aumento exponencial com a idade. Pessoas viúvas, negras, pardas e residentes de áreas rurais apresentaram níveis mais elevados de fragilidade. Os modelos de regressão demonstraram associações entre fragilidade elevada e autoavaliação de saúde inferior, maior incapacidade e maior utilização dos serviços de saúde. Este estudo revela uma alta prevalência de fragilidade em adultos de meia idade e idosos no Brasil, bem como sua associação com a incapacidade, o estado de saúde e o uso dos serviços de saúde. Esses achados são importantes, pois podem orientar as políticas de saúde e o desenho de serviços que priorizem a saúde dessa população, especialmente aqueles que utilizam o sistema público de saúde.

## Resumen

La población anciana de Brasil representará un desafío para los servicios de salud pública, de los cuales depende la mayoría de los brasileños. El país debe prepararse para los desafíos sanitarios relacionados con el envejecimiento, tales como la fragilidad. Los datos del *Estudio Longitudinal de la Salud de los Ancianos Brasileños* (ELSI-Brasil) se utilizaron para generar un Índice de Fragilidad (IF) estandarizado, con el fin de evaluar los niveles de fragilidad en esta población y de proporcionar información confiable a nivel nacional. En la segunda ola de ELSI-Brasil participaron 9.901 adultos de 50 años o más. A partir de un protocolo estandarizado se construyó un FI de 53 ítems. La regresión logística se utilizó para determinar la asociación entre el nivel de fragilidad y la discapacidad/estado de salud, mientras que la regresión binomial negativa analizó la relación entre el nivel de fragilidad, las discapacidades y el uso de los servicios de salud. La fragilidad fue alta, con una puntuación IF promedio ponderada de 0,19 y una mediana de 0,19. La distribución de la fragilidad mostró una asimetría positiva, con niveles más altos en las mujeres y un aumento exponencial con la edad. Las viudas, los negros, los pardos y los residentes de las zonas rurales mostraron niveles más altos de fragilidad. Los modelos de regresión demostraron asociaciones entre alta fragilidad y menor autoevaluación de la salud, mayor discapacidad y mayor uso de los servicios de salud. Se concluye que este estudio revela una alta prevalencia de fragilidad en adultos de mediana edad y ancianos en Brasil, así como su asociación con la discapacidad, el estado de salud y el uso de los servicios de salud. Estos hallazgos son importantes para orientar las políticas de salud y el diseño de servicios que prioricen la salud de esta población, especialmente los usuarios del sistema público de salud.

## Introduction

Frailty, a clinically identifiable state of diminished physiological reserve, results in vulnerability to several adverse health outcomes ^1^ ^,^ ^2^, significantly worrying public health systems, especially due to the growth of the aging population ^3^. The complex interplay of biopsychosocial factors that cross multiple physiological systems contributes to frailty, leading to diminished autonomy in independent activities and causing considerable healthcare expenditures ^4^. These include increased hospitalization and a greater need for intensive care, which ultimately correlate with higher mortality rates ^5^.

Despite its recognized importance, the field lacks a standardized approach to measuring frailty in older Brazilian adults, highlighting the critical gap this study intends to address. The *Brazilian Longitudinal Study of Aging* (ELSI-Brazil, acronym in Portuguese) serves as a pivotal resource in this endeavor, offering insights to refine and apply a standardized Frailty Index (FI) ^6^ ^,^ ^7^. This will facilitate a comprehensive assessment of frailty levels and explore associations with disability, self-reported health, and healthcare use, thereby filling the gap in the literature.

Prior to the COVID-19 pandemic, 2015-2016 data indicated that about 13% of the Brazilian individuals aged 60 years and above were classified as frail based on the frailty phenotype approach ^8^. This figure likely increased after the pandemic due to stressors such as isolation, depression, and anxiety, underscoring the urgency of individually and socially addressing frailty. According to the Brazilian Institute of Geography and Statistics ^9^, the anticipated rise in the Brazilian older adult population from an estimated 16% in 2024 to a projected 34% by 2060 further emphasizes the pressing need for effective frailty management strategies within public health frameworks.

Frailty measurement is fundamental to understanding its impact, preventing its progression and determining opportunities for intervention. Current assessment tools include the Fried physical frailty phenotype (or syndrome) ^10^ and the FI ^11^. FI is notably based on the cumulative health deficits accrued with age, which are more pronounced among those with poorer health ^12^. Although these individual deficits may appear negligible, they produce a substantial collective effect on overall health ^13^. The established reliability of the FI in predicting mortality and adverse outcomes reinforces its utility in clinical practice and research ^14^.

Given the Brazilian demographic shifts and their associated increase in frailty prevalence, this study significantly contributes to public health policymaking and care services for older adults. By providing a standardized FI, assessing frailty levels in this population, and examining relationships with disability, self-reported health, and healthcare use, this study aimed to supply reliable nationwide frailty information and shape strategic public health interventions that effectively meet the demands of the aging population.

## Methods

### The ELSI-Brazil

ELSI-Brazil is a nationally representative survey of individuals aged 50 years or older living in 70 municipalities across five Brazilian mesoregions ^6^ ^,^ ^7^. It aimed to examine the dynamics of aging in the Brazilian population, the factors that influence it, and how the aging population might benefit from social and health services. This information can support policy decisions related to healthy aging and more equitable healthcare access for those in older age groups and enable cross-country comparisons with other similarly aged cohorts For comprehensive details on the methodology of ELSI-Brazil and its national representativeness, readers are encouraged to read Lima-Costa et al. ^6^ ^,^ ^7^, whose the extensive information provides deeper insights into the framework and scope of this significant research initiative. For more information about the ELSI-Brazil study and to submit a request for data access, interested researchers can visit the website: http://elsi.cpqrr.fiocruz.br.

For the first wave of the survey, data were collected from 2015 to 2016 with 9,412 individuals. The second wave occurred from 2019 to 2021, with 9,949 participants, including those selected during sample refreshment. A third wave of the survey was to occur in 2023. This study is a secondary analysis of the data collected during the second wave of the survey (2019-2021).

ELSI-Brazil was approved by the Research Ethics Committee at René Rachou Institute, Oswaldo Cruz Foundation (protocol n. CAAE 34649814.3.0000.5091). All participants provided their consent and signed an informed consent form.

### Variables

Our analysis incorporated several demographic and healthcare-related variables, including sex, age, marital status, ethnicity, area of residence, and medical service providers. Sex was categorized into “female” and “male”. Age was segmented into four groups: “50-64”, “65-79”, “80-94”, and “95+ years”. Marital status was divided into the categories “single”, “married/common-law marriage/live together”, “divorced or separated”, and “widow(er)”. Race was classified into the categories “white”, “black”, “mixed-race”, “yellow/Indigenous”, and “did not know/did not answer”. The area of residence was divided into “rural” and “urban”, and the healthcare provider was categorized as “public”, “private”, and “did not answer”.

### Frailty assessment

An FI was developed based on established procedures and protocols ^15^. The steps to construct the FI are as follows.

#### Item selection

We conducted an in-depth selection for health-related items to determine potential deficits in illnesses, disabilities, and social and mental health issues ^16^. The procedure encompassed a thorough consideration of all applicable health-related factors. Box 1 lists the items included in the FI.

**Box 1:** Items included in the Frailty Index (FI).

#	ITEM	DESCRIPTION
General health and diseases		
1	n1	In general, how would you evaluate your health?
2	n6	How do you evaluate your far vision (EVEN WHEN USING GLASSES OR CONTACT LENSES), that is, recognizing someone that you know on the other side of the street at a distance of 65 feet or so?
3	n7	How do you evaluate your near vision (EVEN WHEN USING GLASSES OR CONTACT LENSES), this means recognizing an object that is within reach or reading a newspaper?
4	n16	How do you evaluate your hearing (EVEN WHEN USING A HEARING DEVICE)?
5	n28	Has any doctor ever told you that you have arterial hypertension (high blood pressure)?
6	n35	Has any doctor ever told you that you have diabetes (“high blood sugar”)?
7	n44	Has any doctor ever told you that you have high cholesterol?
8	n46	Has any doctor ever told you that you had a heart attack?
9	n48	Has any doctor ever told you that you have angina pectoris?
10	n50	Has any doctor ever told you that you have a heart failure?
11	n52	Has a doctor ever told you that you had a cerebral vascular accident (stroke)?
12	n56	Has a doctor ever told you that you have arthritis or rheumatism?
13	n57	Has a doctor ever told you that you have osteoporosis?
14	n60	Has a doctor ever told you that you have or had cancer?
15	n62	Has a doctor ever told you that you have Parkinson’s disease?
16	n63	Has a doctor ever told you that you have Alzheimer’s disease?
17	n69	In the PAST THREE MONTHS, have you lost weight without any dieting?
18	n72	In the LAST WEEK, how often have you felt you could not handle your activities (started something but could not finish it)?
19	n73	In the PAST WEEK, how often have your daily activities required a big effort from you?
20	n74	How would you evaluate the quality of your sleep?
21	n74_1	How often do you have trouble falling asleep (laying down and sleeping)?
22	n74_2	How often do you have trouble sleeping because you wake up during the night?
23	n74_3	How often do you have trouble sleeping because you wake up too early and cannot go back to sleep?
Mobility		
24	p5	Do you have difficulty running or jogging one kilometer or 10 blocks?
25	p6	Do you have difficulty walking one kilometer continuously?
26	p7	Do you have difficulty walking 100 meters (one block)?
27	p8	Do you have difficulty climbing SEVERAL flights of stairs WITHOUT RESTING?
28	p9	Do you have difficulty climbing ONE flight of stairs WITHOUT STOPPING or resting?
29	p10	Do you have difficulty sitting still for about two hours?
30	p10_1	Do you have difficulty getting up from a chair after sitting for a long time?
31	p12	Do you have difficulty bending over, kneeling, or crouching?
32	p13	Do you have difficulty with extending one or both arms above shoulder level?
33	p14	Do you have difficulty with pulling or pushing large objects, such as an armchair?
34	p15	Do you have difficulty with lifting or carrying weights heavier than 5 kilograms, like a heavy bag of groceries?
35	p16	Do you have difficulty picking up a coin from a table? (Cannot drag the coin to pick it up)
Instrumental activities of daily living		
36	p20	Do you have any difficulty preparing A HOT MEAL?
37	p24	Do you have any difficulty USING ANY TYPE OF TRANSPORTATION as a passenger?
38	p26	Do you have any difficulty DOING SHOPPING?
39	p28	Do you have any difficulty USING TELEPHONE (LANDLINE OR CELLULAR)?
40	p30	Do you have any difficulty TAKING/MANAGING YOUR OWN MEDICATIONS?
41	p31_1	Do you have any difficulty WALKING AROUND YOUR HOUSE OR IN THE GARDEN?
42	p22	Do you have any difficulty MANAGING YOUR OWN MONEY?
43	p33	Do you have any difficulty with PERFORMING LIGHT HOUSEKEEPING (making your own bed, removing dust, taking care of the garbage etc.)?
44	p35	Do you have any difficulty PERFORMING HEAVY HOUSEKEEPING?
Basic activities of daily living		
45	p37	Do you have any difficulty GETTING ACROSS A ROOM OR WALKING FROM ONE ROOM TO ANOTHER on the same floor?
46	p40	Do you have any difficulty DRESSING UP?
47	p17	Do you have any difficulty DOING YOUR OWN PERSONAL HYGIENE?
48	p43	Do you have any difficulty SHOWERING?
49	p46	Do you have any difficulty EATING from a dish that was placed in front of you?
50	p49	Do you have any difficulty GETTING IN OR OUT OF BED?
51	p55	Do you have any difficulty USING THE BATHROOM?
52	p58	In the LAST MONTH, have you ever unintentionally lost control of urine?
53	p58	In the LAST MONTH, have you ever unintentionally lost control of feces?

#### Coding

The selected variables were transformed into binary scales (0 and 1), meaning “no deficit” and “deficit”, respectively. In cases in which the variables had more than two possible answers, the deficit was calculated as a fraction of the total deficit. For exemple, self-rated health had six potential responses: excellent (0), very good (0.2), good (0.4), fair (0.6), poor (0.8), and very poor (1).

#### Exploratory analysis

All the items were screened for gaps in the data and how they were associated with age. Items with a prevalence lower than one percent and those with more than five percent missing values were removed. Spearman’s correlation coefficient was computed to evaluate the correlation between each item and age. When the correlation with age was equal to or lower than 0.01, the item was removed from the FI as it offered no meaningful data. Moreover, all participants with more than 20% of missing deficits were excluded.

#### FI calculation

To calculate the FI score for each participant, the total number of identified deficits was added and then divided by the total number of deficits. Scores ranged from 0 to 1, in which zero indicated the lowest possible frailty and one, all deficits ^17^. Practice obtains a subclinical limit to the degree of frailty at about 0.7, reflecting the lethality of deficit accumulation ^17^ ^,^ ^18^.

#### Explorations of FI

The robustness of an FI based on population data can be examined by several key properties. First, as age increases, so does the FI. Second, FI usually has a gamma distribution in population-based samples (i.e., right-skewed), and is generally expected to be higher for women than for men ^11^ ^,^ ^18^. These characteristics were investigated and described in the Supplementary Material (https://cadernos.ensp.fiocruz.br/static//arquivo/suppl-e00041624_9157.pdf).

### Disability, self-reported health, and healthcare use

Disability was assessed based on basic activities of daily living (BADLs) and instrumental activities of daily living (IADLs). BADLs pertain to basic tasks such as walking, getting dressed, showering, eating, and using the bathroom, whereas IADLs refer to more complex activities, such as preparing a hot meal, managing finances, using transportation, and shopping ^19^ ^,^ ^20^. A detailed explanation of the daily living activity assessment questions is provided in the Supplementary Material (https://cadernos.ensp.fiocruz.br/static//arquivo/suppl-e00041624_9157.pdf).

Self-reported health was evaluated according to participants’ answers to the question, “In general, how would you evaluate your health?”. Self-reported health was scored high if participants answered “excellent”, “very good”, or “good”, and low if, “very poor”, “poor”, or “fair”.

Healthcare use was determined by tracking the number of times an individual visited a healthcare provider (e.g., a doctor or specialist) and the number of times they were admitted to a hospital in the 12 months prior to this research.

### Statistical analyses

To generate population-representative estimates of FI, the *survey* package on R was used, which incorporates the ELSI-Brazil weighting and sampling design. These estimates are shown as weighted means and 95% confidence intervals (95%CI). Moreover, frailty levels were shown for the overall population and sociodemographic subsets (i.e., by age group, marital status, race, area of residence, and healthcare provider).

The relative frequency distribution of the FI was graphically represented by a histogram. Several regression models (including linear, cubic, and exponential ones) were examined to determine the most suitable fit to measure the relationship between age and the FI. The FI logarithm was then calculated and regressed against age to analyze the correlation between these two values. Exponential predictions were used to obtain the FI for men and women of different ages.

The frequency of disabilities in IADLs and BADLs were analyzed as distinct categories. To avoid any overlap between indicators of disability and the measure of frailty, IADL and BALD self-reported items were excluded from the FI during the regression analyses for each respective measure. Logistic regression models were used to explore the relation between the FI and disability considering age, sex, race, marital status, and education as adjustments. This model calculated the value of FI, which indicated a 50% probability of at least one disability. Additionally, a negative binomial regression was conducted to investigate the relation between frailty and number of disabilities. This model was adjusted for age, sex, race, marital status, and education, and the number of disabilities was modelled as a function of FI. Results are shown as a prediction plot that covered a range of FI values and an incidence risk ratio (IRR) table. FI values are also described corresponding to at least one disability following this model. Finally, the proportion of individuals with disabilities was calculated based on their frailty levels. The Mann-Kendall test was used to evaluate trends in the association between frailty groups and categorical proportions.

The data on self-reported health were analyzed by plotting the frequency of responses. To avoid any potential overlap between indicators of self-reported health and the measure of frailty, self-reported health items were excluded from the FI during the regression analyses for each measure. How individuals rated their health was examined and the data were analyzed by a logistic regression model to determine the relationship between health status and FI. Age, sex, race, marital status, education, and disabilities related to IADL and BADL were considered as covariates in the adjustment process.

How often people had visited doctors, specialists, and hospitals in the past year for healthcare use was determined. A negative binomial regression model was employed, with age, sex, race, marital status, education, and disability included as covariates. This analysis enabled the estimation of visit and hospitalization counts as a function of FI. The proportion of visits and hospitalizations was examined based on frailty levels, using the Mann-Kendall test to analyze trends in the association between frailty groups and categorical proportions.

All analyses were conducted on R, version 4.4.0 “Puppu Cup” release (http://www.r-project.org) and RStudio IDE, 2024.04.0+735 “Chocolate Cosmos” release (https://rstudio.com/).

## Results

### Frailty

After reviewing 88 health-related items from the ELSI-Brazil second-wave dataset, we excluded 14 items due to more than 5% missing values and one item with a low prevalence (below 1%). This study also excluded an additional 20 items due to their weak correlation with age (less than 0.01). We then selected 53 items for the FI analysis (Box 1 and the Supplementary Material; https://cadernos.ensp.fiocruz.br/static//arquivo/suppl-e00041624_9157.pdf). Moreover, three additional FIs aimed to examine the relationship between FI, disability, and self-reported health status. The first version excluded IADL items; the second one, IADL and BADL items; and the third one, self-reported questions.

**Figure 1:**
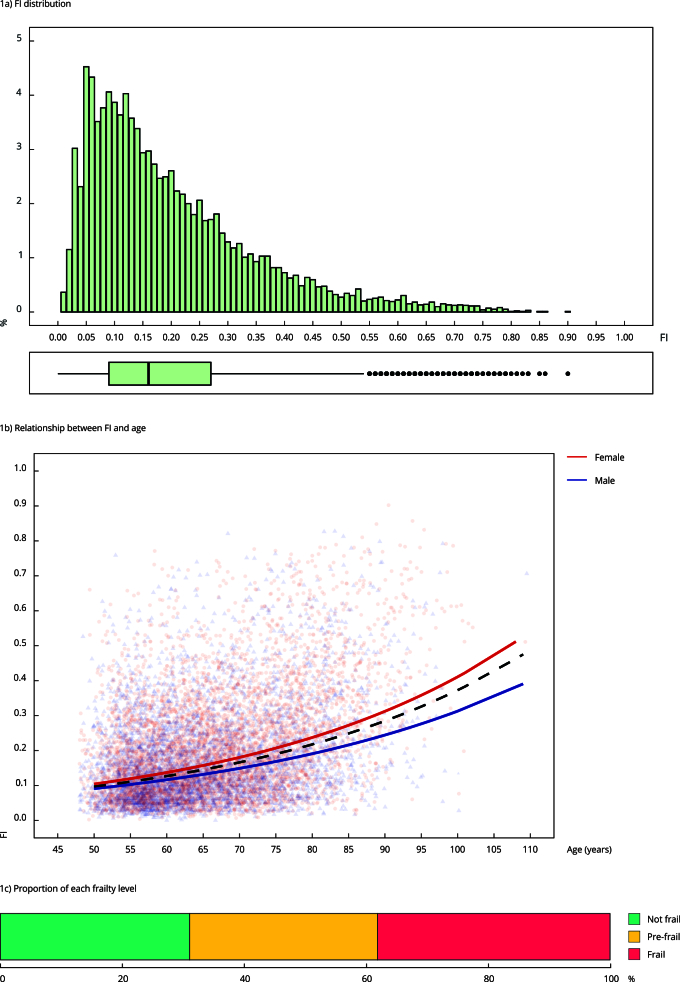
The Frailty Index (FI) distribution is shown in the histogram, whereas the scatterplot displays the relationship between FI and age with both exponential (dashed black line) and sex-specific models (solid red and blue lines). The stacked bar illustrates the proportion of each frailty level determined by the pre-established FI cut-offs.

The second wave of the ELSI-Brazil survey included 9,949 Brazilian adults who were aged ≥ 50 years. However, this analysis excluded 48 individuals because of significant missing data that exceeded 20% of the observed variables. This analysis included 9,901 participants; its weighted adjustment represented 53,865,660 middle-aged and older Brazilians. The weighted mean age equaled 63 ± 10 years, and 50.1% of the respondents were women. The weighted mean FI score of the sample totaled 0.19, with a standard deviation of 0.14 (Table 1). Women and older individuals had higher FI scores. About 0.1% of the sample had an FI score of zero; 99.4%, an FI score ≤ 0.7; and the maximum recorded score equaled 0.90 (< 0.01%). The weighted median FI score totaled 0.15 (1st-3rd quartiles, 0.08-0.25), and the distribution was slightly skewed to the right (Figure 1a).

**Table 1:** Weighted * descriptive statistics estimating the values of the participants in the 2019-2022 *Brazilian Longitudinal Study of Aging* (ELSI-Brazil) (N = 9,901).

Variables	Weighted frequency		Weighted mean FI (95%CI)
	n	% (95%CI)	
Overall	53,865,660	100.0	0.19 (0.18-0.20)
Sex			
Female	26,964,797	50.1 (47.0-53.0)	0.21 (0.20-0.22)
Male	26,900,864	49.9 (47.0-53.0)	0.17 (0.16-0.18)
Age group (years)			
50-64	33,627,815	62.4 (59.0-66.0)	0.16 (0.15-0.17)
65-79	16,182,013	30.0 (28.0-33.0)	0.21 (0.20-0.22)
80-94	3,919,289	7.3 (6.2-8.5)	0.33 (0.31-0.35)
95+	136,544	0.3 (0.17-0.37)	0.47 (0.40-0.53)
Marital status			
Single	6,805,668	12.6 (11.0-14.0)	0.19 (0.18-0.21)
Married/Common-law marriage/Living together	32,693,576	60.7 (57.0-64.0)	0.17 (0.17-0.18)
Divorced or separated	5,973,338	11.1 (9.9-12.0)	0.16 (0.15-0.18)
Widow(er)	8,393,078	15.6 (14.0-17.0)	0.25 (0.23-0.27)
Race			
White	24,924,436	46.3 (40.0-52.0)	0.18 (0.17-0.19)
Black	5,810,490	10.8 (8.6-13.0)	0.21 (0.19-0.24)
Mixed-race	22,700,648	42.1 (37.0-47.0)	0.19 (0.18-0.20)
Yellow/Indigenous	251,534	0.5 (0.28-0.77)	0.18 (0.13-0.23)
Did not know/Did not answer	178,553	0.3 (0.20-0.55)	0.15 (0.10-0.19)
Area of residence			
Rural	8,423,682	15.6 (11.0-22.0)	0.18 (0.17-0.20)
Urban	45,441,978	84.4 (78.0-89.0)	0.19 (0.18-0.20)
Healthcare provider			
Public	43,212,858	80.2 (77.0-83.0)	0.19 (0.18-0.20)
Private	10,569,628	19.6 (17.0-23.0)	0.18 (0.16-0.19)
Did not answer	83,174	0.2 (0.09-0.27)	0.14 (0.08-0.21)

95%CI: 95% confidence interval; FI: Frailty Index.

* The ELSI-Brazil survey employs a weighting and sampling design to produce estimates that accurately reflect the population of Brazilians aged 50 and above.

Additionally, log-FI increased exponentially with age, rising to 0.026 each year (Figure 1b). Women consistently showed higher FI scores than men across all age categories. Figure 1c illustrates the distribution of frailty levels based on predetermined FI cut-off values.

The Supplementary Material (https://cadernos.ensp.fiocruz.br/static//arquivo/suppl-e00041624_9157.pdf) shows detailed prevalence estimates of frailty levels according to sociodemographic characteristics and age groups. These results show differences in the distribution of frailty levels based on sociodemographic factors since widow(er)s, black and mixed-race individuals, and those living in rural areas have higher levels of frailty.

### Frailty and disability

We observed that more than 20% (95%CI: 17-24) reported at least one IADL disability. The findings of the logistic regression analysis indicated that the FI, when constructed to exclude IADL-related items, was associated with at least one IADL disability status (adjusted for age, sex, race, marital status, and education; p < 0.001). Moreover, the model suggested that an FI (excluding IADL items) of 0.30 corresponds to a 50% likelihood of experiencing at least one disability in the IADL. An FI score of 0.40 yielded a 2-fold increase in the prevalence of two or more IADL disabilities. The negative binomial model showed a significant association between higher frailty levels and an increase in the number of disabilities. This trend persisted even after considering factors such as age, sex, race, marital status, and education. The trend peaked when the index exceeded 0.30 (with a p-value for a trend of 0.008). The maximum number of disabilities occurred at a FI of 0.53 (see Supplementary Material for more details; https://cadernos.ensp.fiocruz.br/static//arquivo/suppl-e00041624_9157.pdf).

About 10.7% (95%CI: 8.9-13.0) of individuals reported at least one BADL disability. Even excluding IADL- and BADL-related items from FI, results showed that frailty was linked to at least one BADL disability (adjusted for age, sex, race, marital status, and education; p < 0.001). Based on our model, an FI score of 0.34 corresponds to a 50% probability of facing at least one disability in BADL. Once again, an FI score of 0.40 yielded a 2-fold increase in the prevalence of two or more BADL disabilities. The negative binomial model also showed an association between higher frailty levels and an increased number of disabilities. This trend persisted even after considering factors such as age, sex, race, marital status, and education. The trend peaked when the index score exceeds 0.30 (with a p-value for a trend of 0.008). The maximum number of disabilities occurred at an FI of 0.47 (see Supplementary Material for more details; https://cadernos.ensp.fiocruz.br/static//arquivo/suppl-e00041624_9157.pdf).

### Frailty and self-perceived health status

Overall, participants reported a low self-perceived health status. A significant 52.1% of them (95%CI: 49.0-55.0) reported low self-perception of their health status. After adjusting it for age, sex, race, marital status, education, and at least one IADL or BALD disability, the logistic regression analysis showed that the frailty level was independently associated with self-rated health status. Individuals with higher levels of frailty were more likely to report lower self-rated health status (see Supplementary Material for more details; https://cadernos.ensp.fiocruz.br/static//arquivo/suppl-e00041624_9157.pdf).

### Frailty and healthcare service use

Most participants (72.5%) stated that they had visited a physician at least once in the year prior to this research. According to the negative binomial model adjusted for age, sex, race, marital status, and education, higher FI scores were associated with more visits to doctors in the 12 months prior. IRR totaled 8.59 (95%CI: 7.29-10.13; p < 0.001). Once the FI reached 0.17, the number of health consults over the past 12 months became more apparent.

Of those seeking medical advice in the year prior, 65.1% consulted a specialist. Among these, 66.6% had more than two consultations with a specialist in the same period. According to the negative binomial model adjusted for age, sex, race, marital status, and education, a higher FI indicated more specialist visits in the past year. IRR equaled 2.95 (95%CI: 2.50-3.48; p < 0.001). This became more noticeable when the FI score totaled 0.11 due to the increase in the number of consultations with specialists in the 12 months prior to this research.

Moreover, about 6% of respondents stated having been admitted to the hospital once or more in the past year. Furthermore, the frequency of hospitalization within the past year was associated with the degree of frailty. When the FI score reached 0.72, hospitalization was considered universal (see Supplementary Material for more details; https://cadernos.ensp.fiocruz.br/static//arquivo/suppl-e00041624_9157.pdf).

## Discussion

This study used a secondary analysis of the ELSI-Brazil dataset to generate standardized information about frailty in the Brazilian population. Results showed that FI had the expected characteristics, including a right-skewed distribution, higher levels of frailty in women than in men, and an exponential increase with age. We found a high frailty, with a 0.19 weighted mean FI and a 0.15 median. The established cut-offs deemed an FI of ≥ 0.21 as frail ^21^. Moreover, widow(er)s, black and mixed-race individuals, and those living in rural areas had higher levels of frailty. Regression models showed that higher frailty levels were significantly associated with poorer self-rated health status and higher healthcare utilization rates. The results of this study provide valuable insights into the prevalence and distribution of frailty levels in the Brazilian population and suggest the need for a better understanding of the factors that contribute to frailty and its associated health outcomes.

Frailty phenotype and FI can accurately predict adverse outcomes, despite the variable magnitude of the effect ^22^. A 2018 study using data from the first-wave ELSI-Brazil and frailty phenotype estimated that 9% of adults aged ≥ 50 years in Brazil as frail, increasing to 20.9% in those aged ≥ 70 years ^8^. However, our study showed a heightened degree of frailty, classifying about 38% of individuals as frail. This difference could be attributed to the data collection for the ELSI-Brazil second wave occurring during the COVID-19 pandemic and the use of the FI method for the first time to assess frailty in the Brazilian population aged 50 years or older as opposed to the frailty phenotype.

A recent systematic review of community-dwelling adults showed that the prevalence of frailty ranged from 4% to 59%, mainly because of unlike definitions of frailty ^3^. Our group has shown varying rates of frailty in the *National Health and Nutrition Examination Survey* in the United States depending on the method: the modified frailty phenotype estimated a 3.6% prevalence, whereas the FI, one of 34% ^23^. Despite the convergence in predicting adverse clinical outcomes, differences remain between the phenotypic definitions of frailty and FI. Broadly speaking, the phenotypic definition offers ready clinical operationalization to distinguish general levels of risk, whereas FI can more precisely define risk ^22^ and granularity in population data.

This study observed a clear association between frailty and disability. About 10% of participants had at least one BADL disability and 20%, an IADL disability. These numbers were particularly evident in individuals whose frailty levels exceeded 0.30 (more so after 0.40 in which prevalence considerably exceeded that of other frailty levels). The relationship between frailty and disability is notably complex as levels of disability often lead to increased morbidity and worsening of existing medical conditions ^24^ ^,^ ^25^. Furthermore, frailty may affect function, which could signify its presence. Notably, although the experience of disability increased at higher levels of frailty (even when building the FI excluded IADL and BADL items), this correlation remained imperfect. This supports the view that frailty and disability are related and yet distinct constructs. Ultimately, frailty should not simply be regarded as a pre-disability stage ^23^ ^,^ ^26^.

Recent investigations into the effect of frailty on healthcare expenditure and use have evinced a definite pattern: a higher degree of frailty is associated with higher costs and greater healthcare use ^3^. Our research reaffirmed the relationship between FI, decreased self-perceived health status, and increased healthcare utilization, expanding it to a nationally representative level. By expanding the knowledge on the correlation between older adults with frailty and healthcare services in Brazil (an upper-middle-income country), this study sheds light on how frailty can affect local healthcare systems, particularly its Brazilian Unified National Health System. Our research indicates that most middle-aged and older individuals in Brazil (about 80%) depend on the public healthcare system. This is a crucial finding, as our data and nationwide statistics from to 2015-2016 show that older adults aged 60 years and above who rely on public healthcare services are more likely to experience greater frailty than those who choose private healthcare ^27^. Moreover, analyzing nationwide data from to 2015-2016 shows that older adults who depend on public healthcare face more challenges in accessing healthcare and receiving continuous care and comprehensive care ^27^.

In contrast, those affiliated with private healthcare plans tend to experience issues mainly related to the comprehensiveness of their coverage ^27^. Considering the far-reaching implications of differences in the profiles of public and private health service users, our results can assist the development of more effective policies and services for older adults living with frailty in Brazil. For example, plan public healthcare services must optimize care for the high levels of user frailty and the expected increase in frailty in the coming years. This is important because it can influence the quality of healthcare individuals receive. Ultimately, this planning positively impacts the overall well-being of the Brazilian older adult population.

Our study has some limitations. Potential biases and inaccuracies may stem from self-reporting bias and memory recall errors, which affect data reliability. Third-party data reliability remains a concern and a methodological constraint. Despite these challenges, self-reported data are indispensable in broad epidemiological studies, although the findings should be interpreted with caution. This study used self-reported health variables and included some gaps in data depending on the variable. Notably, it excluded variables with > 5% gaps from the FI and participants with > 20% missing frailty items. Although ELSI-Brazil broadly represents Brazilian adults aged 50 years and over, certain populations will likely be undersampled due to the sampling frame and inclusion/exclusion criteria, including those living in institutions and in Indigenous and remote communities. This study still provides essential nationwide information on frailty in Brazil among those aged ≥ 50 years. Results may improve healthcare policies and services for older Brazilian adults, particularly those relying on public health care. A strength of the study includes its focus on a population as young as 50 years of age rather than the usual 65+ demographic. This helps us comprehend frailty from a life course point of view, showing that, rather than an instant occurrence, it results from health declines over time. The information in this report is essential to establish appropriate healthcare plans and services for middle-aged and older adults in Brazil, especially those who depend on public healthcare.

In conclusion, this study emphasizes the urgent challenge of frailty among Brazilian middle-aged and older adults, showing that 38% of individuals aged 50 and over are frail, exceeding the numbers in previous estimates and suffering the exacerbation of the COVID-19 pandemic and differing assessment methods. This underscores the significant impact of frailty on public health, which is linked to poorer self-rated health and increased health care use. These insights call for strategic planning to enhance the well-being of older adults in Brazil, ensuring an improved individual quality of life and a more resilient public health system.

## Data Availability

Data and documentation are freely available to researchers upon registration with the ELSI-Brazil project at https://elsi.cpqrr.fiocruz.br/en/register/.

## References

[1] Kim DH, Rockwood K (2024). Frailty in older adults. N Engl J Med.

[2] Travassos GF, Coelho AB, Arends-Kuenning MP (2020). The elderly in Brazil demographic transition, profile, and socioeconomic condition. Rev Bras Estud Popul.

[3] Hoogendijk EO, Afilalo J, Ensrud KE, Kowal P, Onder G, Fried LP (2019). Frailty implications for clinical practice and public health. Lancet.

[4] Taylor JA, Greenhaff PL, Bartlett DB, Jackson TA, Duggal NA, Lord JM (2023). Multisystem physiological perspective of human frailty and its modulation by physical activity. Physiol Rev.

[5] Clegg A, Young J, Iliffe S, Rikkert MO, Rockwood K (2013). Frailty in elderly people. Lancet.

[6] Lima-Costa MF, de Melo Mambrini JV, Bof de Andrade F, de Souza PRB, de Vasconcellos MTL, Neri AL (2023). Cohort profile: The Brazilian Longitudinal Study of Ageing (ELSI-Brazil).. Int J Epidemiol.

[7] Lima-Costa MF, de Andrade FB, de Souza PRB, Neri AL, Duarte YAO, Castro-Costa E (2018). The Brazilian Longitudinal Study of Aging (ELSI-Brazil): objectives and design.. Am J Epidemiol.

[8] Andrade JM, Duarte YAO, Alves LC, Andrade FCD, Souza PRB, Lima-Costa MF (2018). Frailty profile in Brazilian older adults ELSI-Brazil. Rev Saúde Pública.

[9] Instituto Brasileiro de Geografia e Estatística (2024). Projeções da população do Brasil e Unidades da Federação: 2000-2070.

[10] Fried LP, Tangen CM, Walston J, Newman AB, Hirsch C, Gottdiener J (2001). Frailty in older adults evidence for a phenotype. J Gerontol A Biol Sci Med Sci.

[11] Mitnitski AB, Mogilner AJ, Rockwood K (2001). Accumulation of deficits as a proxy measure of aging. ScientificWorldJournal.

[12] Mitnitski A, Rockwood K (2015). Aging as a process of deficit accumulation its utility and origin. Interdiscip Top Gerontol.

[13] Rockwood K, Mitnitski A (2011). Frailty defined by deficit accumulation and geriatric medicine defined by frailty. Clin Geriatr Med.

[14] Kaskirbayeva D, West R, Jaafari H, King N, Howdon D, Shuweihdi F (2023). Progression of frailty as measured by a cumulative deficit index a systematic review. Ageing Res Rev.

[15] Theou O, Haviva C, Wallace L, Searle SD, Rockwood K (2023). How to construct a frailty index from an existing dataset in 10 steps. Age Ageing.

[16] Theou O, Walston J, Rockwood K (2015). Operationalizing frailty using the frailty phenotype and deficit accumulation approaches. Interdiscip Top Gerontol Geriatr.

[17] Rockwood K, Mitnitski A (2006). Limits to deficit accumulation in elderly people. Mech Ageing Dev.

[18] Mitnitski A, Song X, Skoog I, Broe GA, Cox JL, Grunfeld E (2005). Relative fitness and frailty of elderly men and women in developed countries and their relationship with mortality. J Am Geriatr Soc.

[19] Katz S, Ford AB, Moskowitz RW, Jackson BA, Jaffe MW (1963). Studies of illness in the aged The index of ADL: a standardized measure of biological and psychosocial function. JAMA.

[20] Lawton MP, Brody EM (1969). Assessment of older people self-maintaining and instrumental activities of daily living. Gerontologist.

[21] Hoover M, Rotermann M, Sanmartin C, Bernier J (2013). Validation of an index to estimate the prevalence of frailty among community-dwelling seniors. Health Rep.

[22] Rockwood K, Andrew M, Mitnitski A (2007). A comparison of two approaches to measuring frailty in elderly people. J Gerontol A Biol Sci Med Sci.

[23] Blodgett J, Theou O, Kirkland S, Andreou P, Rockwood K (2015). Frailty in NHANES comparing the frailty index and phenotype. Arch Gerontol Geriatr.

[24] Liu HX, Ding G, Yu WJ, Liu TF, Yan AY, Chen HY (2019). Association between frailty and incident risk of disability in community-dwelling elder people evidence from a meta-analysis. Public Health.

[25] Kojima G (2017). Frailty as a predictor of disabilities among community-dwelling older people a systematic review and meta-analysis. Disabil Rehabil.

[26] Theou O, Rockwood MR, Mitnitski A, Rockwood K (2012). Disability and co-morbidity in relation to frailty how much do they overlap?. Arch Gerontol Geriatr.

[27] Silva AMM, Mambrini JVM, Andrade JM, Andrade FB, Lima-Costa MF (2021). Fragilidade entre idosos e percepção de problemas em indicadores de atributos da atenção primária à saúde resultados do ELSI-Brasil. Cad Saúde Pública.

