# Supplementary material for: Exploring frailty in Brazil: an analysis of the ELSI-Brazil survey
Source: Cad Saude Publica. 2025 Apr 28;41(3):e00041624. doi: 10.1590/0102-311XEN041624 (PMC12055010; doi:10.1590/0102-311XEN041624)
Supplement: Supplementary file 1 [file 1678-4464-csp-41-03-EN041624-s.pdf]

SUPPLEMENTARY MATERIAL

This supplementary material provides supporting content for the findings presented in "Exploring Frailty in Brazil: An Analysis of the ELSI-Brazil Survey." Using the ELSI-Brazil dataset, the analysis investigates the prevalence and distribution of frailty levels among the Brazilian population, examines its relationships with disability, self-reported health, and healthcare utilization, and supplies reliable and nationwide frailty information.

Table of Contents

Prevalence estimates of frailty levels 2
FI and IADL disability 7
FI and BADL disability 9
Self-assessed health status 11
Healthcare use: doctor visits 12
Healthcare use: specialist visits 13
Healthcare use: hospitalizations 14
Frailty and healthcare use 15

Prevalence estimates of frailty levels

(A) By age groups

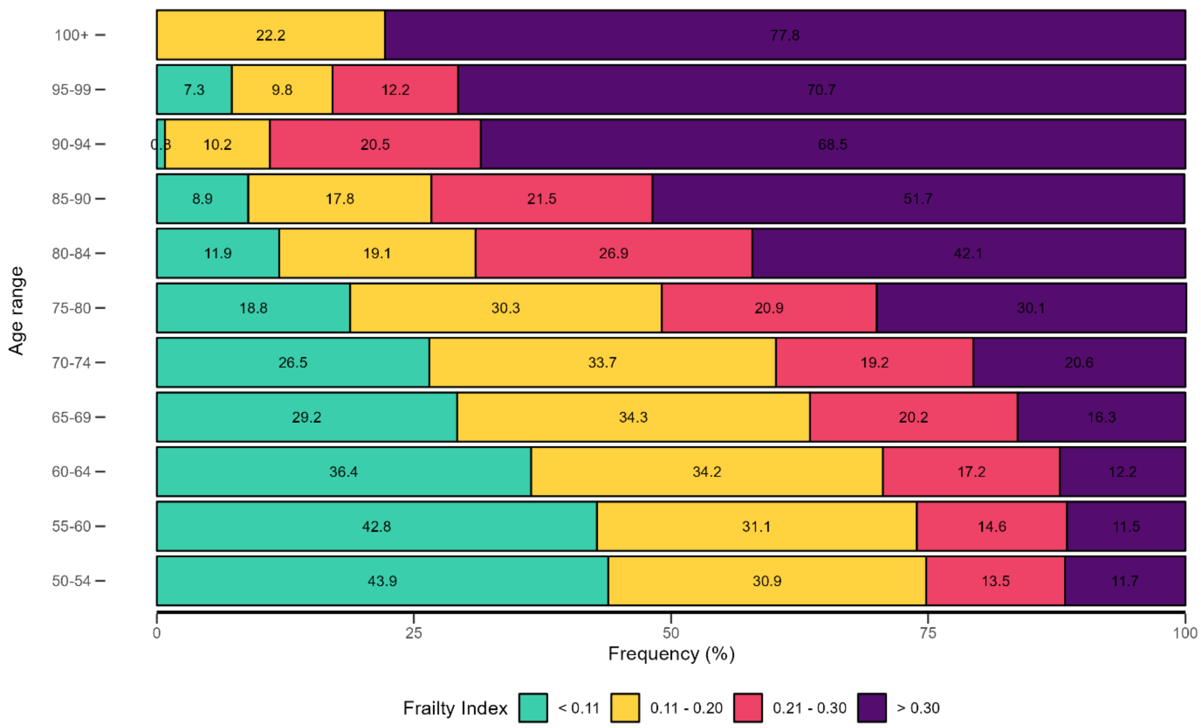

(B) By marital status

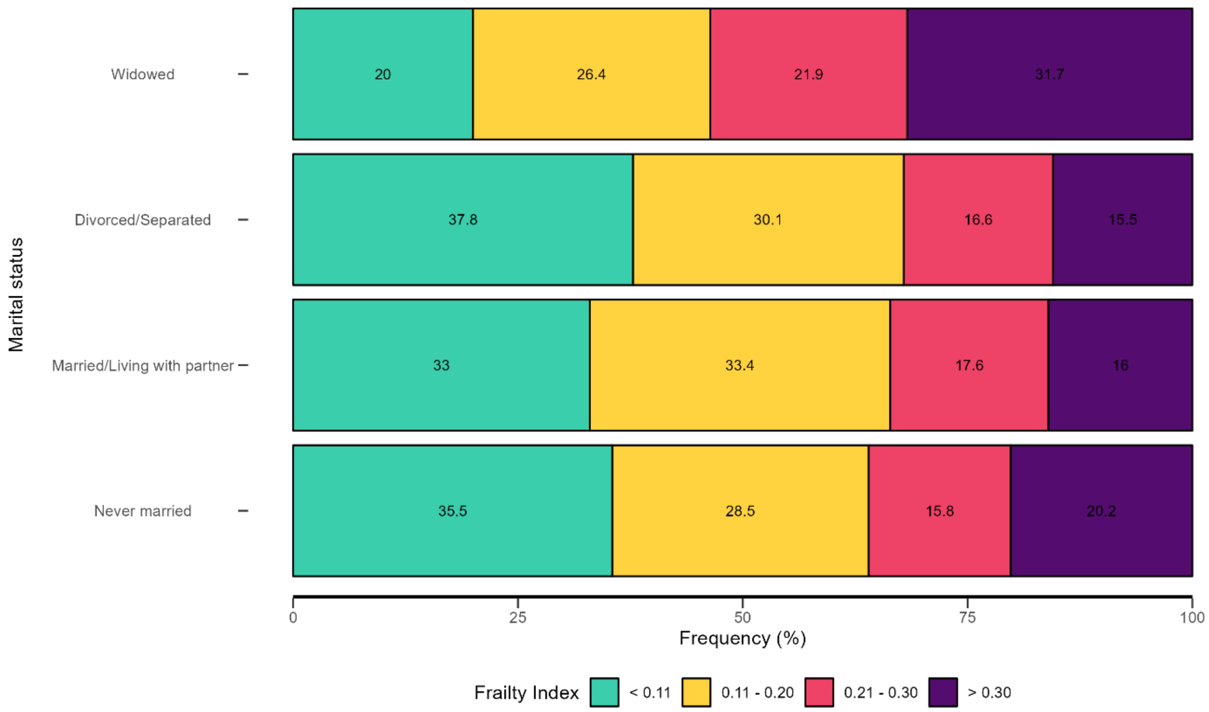

(C) By race

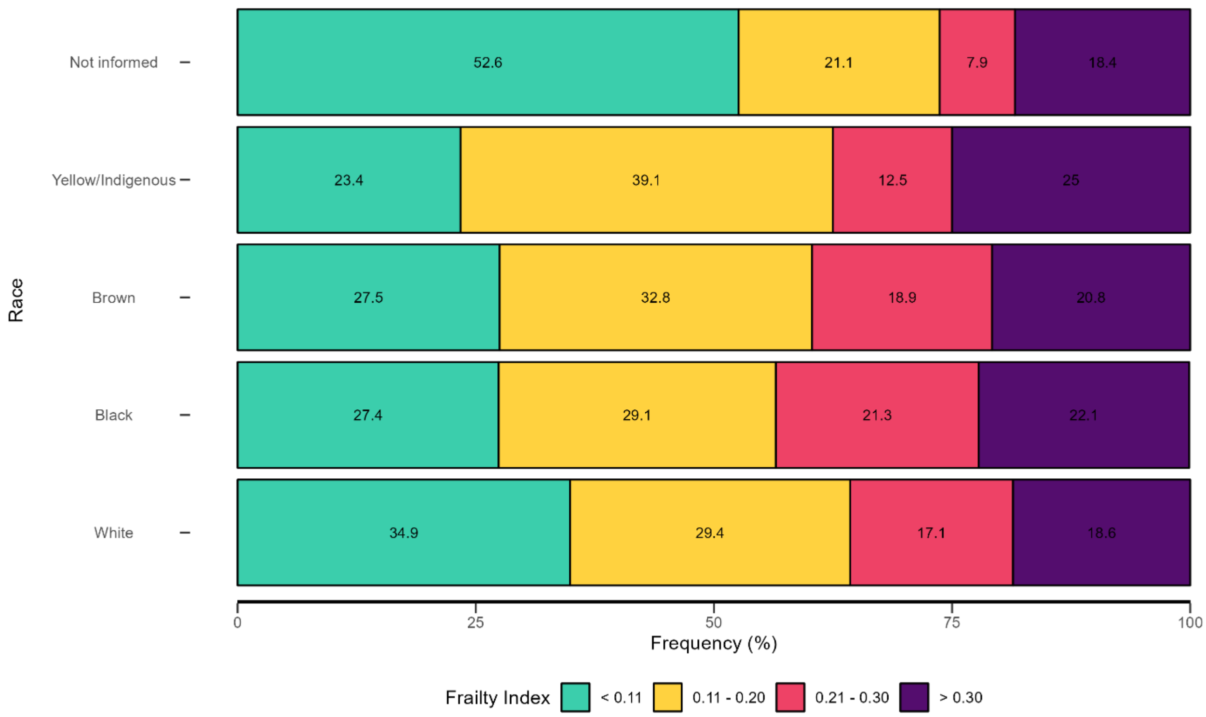

Not informed
Yellow/Indigenous
Brown
Black
White
Race
52.6
21.1
7.9
18.4
23.4
39.1
12.5
25
27.5
32.8
18.9
20.8
27.4
29.1
21.3
22.1
34.9
29.4
17.1
18.6
0
25
50
75
100
Frequency (%)
Frailty Index
< 0.11
0.11 - 0.20
0.21 - 0.30
> 0.30

(D) By area of residence

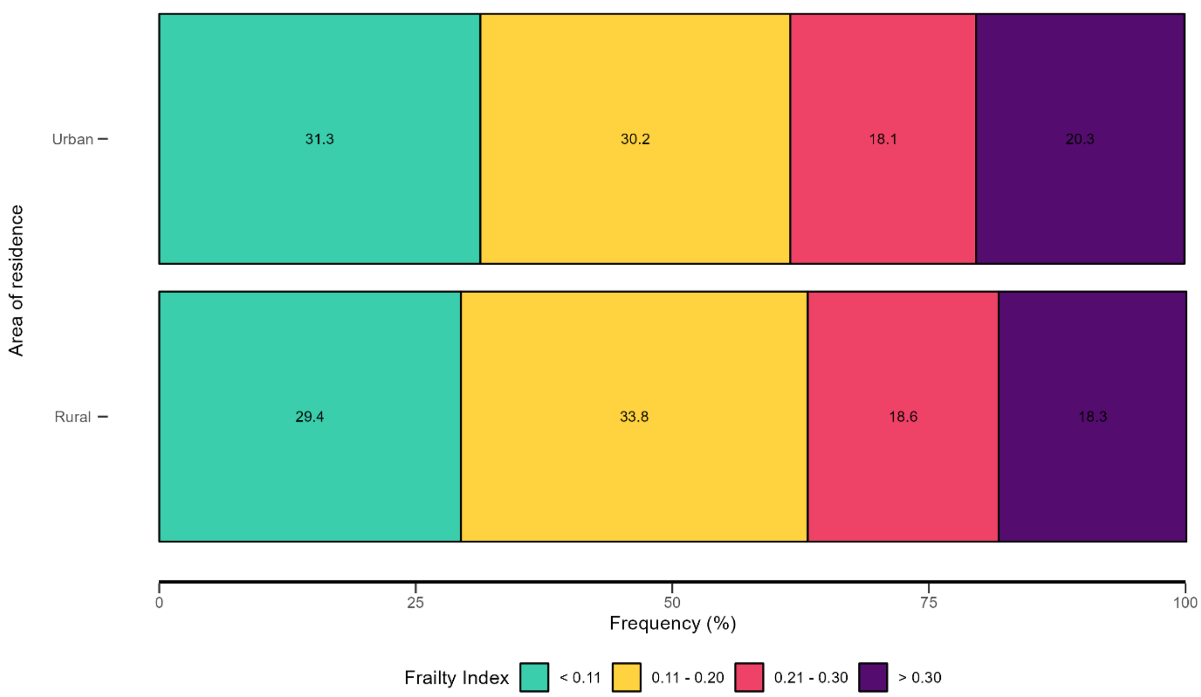

(E) By healthcare provider

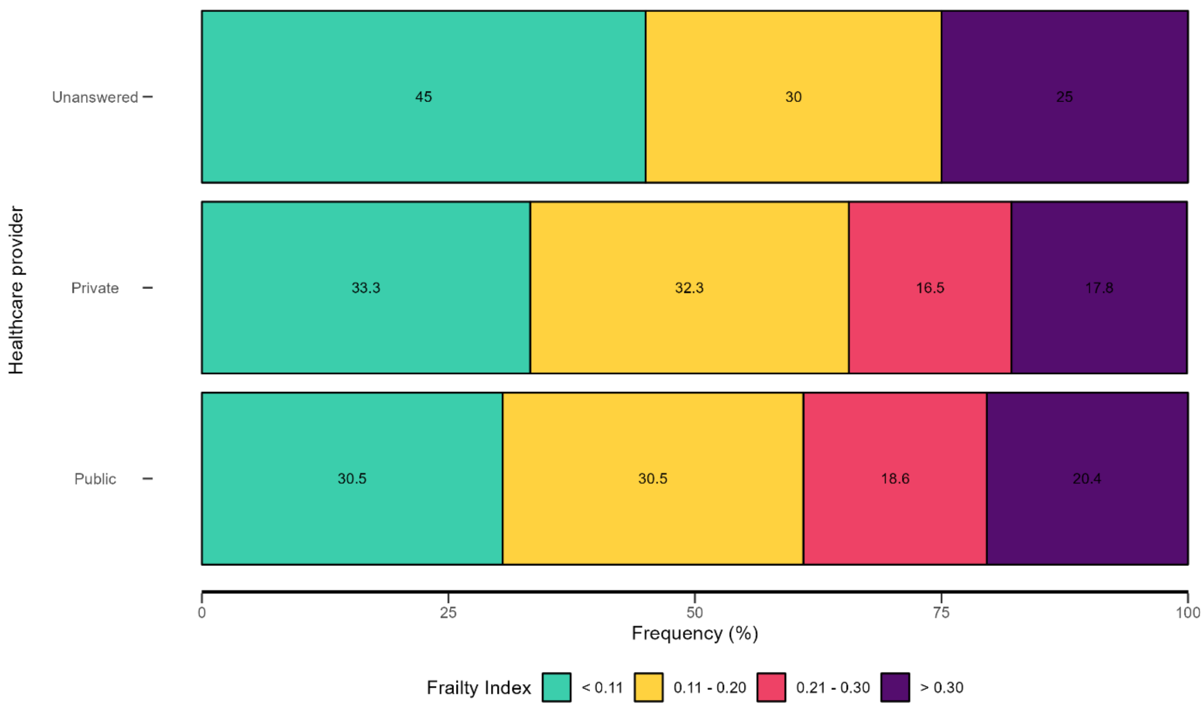

FI and IADL disability

To examine the relationship between FI and IADL disability, the FI was established without taking IADL-related items into account. In Figure A, the estimated prevalence of disabilities in IADL can be seen. Meanwhile, Figure B depicts the estimated probability of having at least one IADL disability based on the frailty index, adjusted for age, sex, race, marital status, and education (Logistic Regression model). The frailty index value corresponding to a 50% probability of having at least one IADL disability is represented by the red dashed line. Figure C displays the frailty index values equivalent to having at least one IADL disability or all disabilities assessed, adjusted for age, sex, race, marital status, and education (Negative Binomial model). Lastly, prevalence estimates for IADL disabilities based on frailty levels are provided in Figure D.

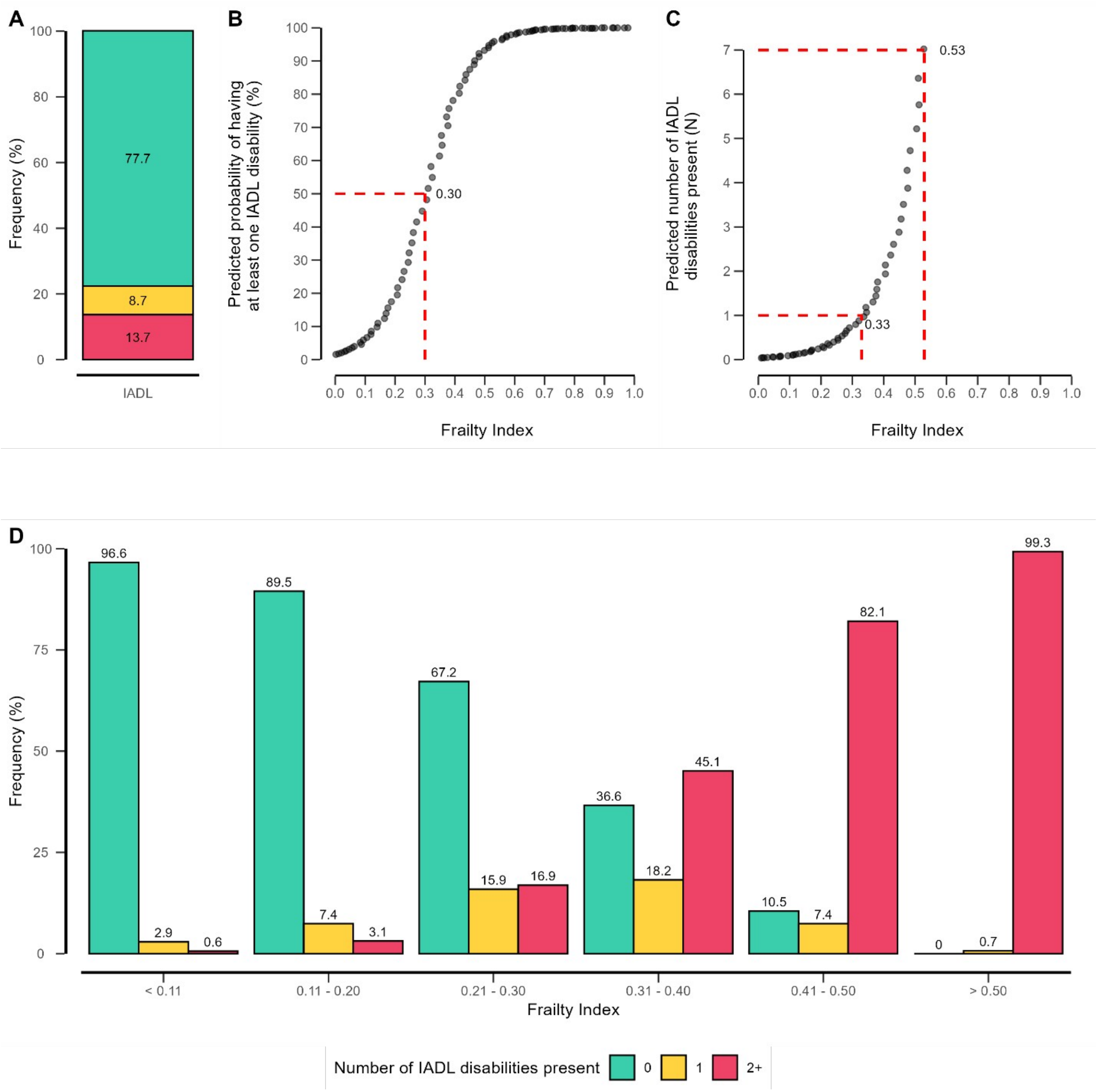
A
B
C
D
Frequency (%)
IADL
77.7
8.7
13.7
Predicted probability of having at least one IADL disability (%)
Predicted number of IADL disabilities present (N)
0.30
0.33
0.53
Frailty Index
96.6
2.9
0.6
89.5
7.4
3.1
67.2
15.9
16.9
36.6
18.2
45.1
10.5
7.4
82.1
0
0.7
99.3
< 0.11
0.11 - 0.20
0.21 - 0.30
0.31 - 0.40
0.41 - 0.50
> 0.50
Number of IADL disabilities present
0
1
2+

To examine the relationship between FI and IADL disability, the FI was established without taking IADL-related items into account. Regressing the count of IADL disabilities to frailty level, adjusted for age, sex, race, marital status, and education.

**Parameter**	**IRR (95% CI) ***	**p**
Frailty Index		
< 0.11	Reference	-
0.11 - 0.20	3.12 (2.58 – 3.78)	< 0.001
0.21 - 0.30	11.71 (9.79 – 14.10)	< 0.001
0.31 - 0.40	28.70 (23.91 – 34.68)	< 0.001
0.41 - 0.50	59.51 (48.66 – 73.23)	< 0.001
> 0.50	76.33 (60.10 – 97.62)	< 0.001

* IRR, Incidence Rate Ratio; 95% CI, 95% Confidence Interval.

FI and BADL disability

To examine the relationship between FI and BADL disability, FI was established without taking IADL and BALD-related items into account. In Figure A, the estimated prevalence of disabilities in BADL can be seen. Meanwhile, Figure B depicts the estimated probability of having at least one BADL disability based on the frailty index, adjusted for age, sex, race, marital status, and education (Logistic Regression model). The frailty index value corresponding to a 50% probability of having at least one IADL disability is represented by the red dashed line. Figure C displays the frailty index values equivalent to having at least one IADL disability or all disabilities assessed, adjusted for age, sex, race, marital status, and education (Negative Binomial model). Lastly, prevalence estimates for BADL disabilities based on frailty levels are provided in Figure D.

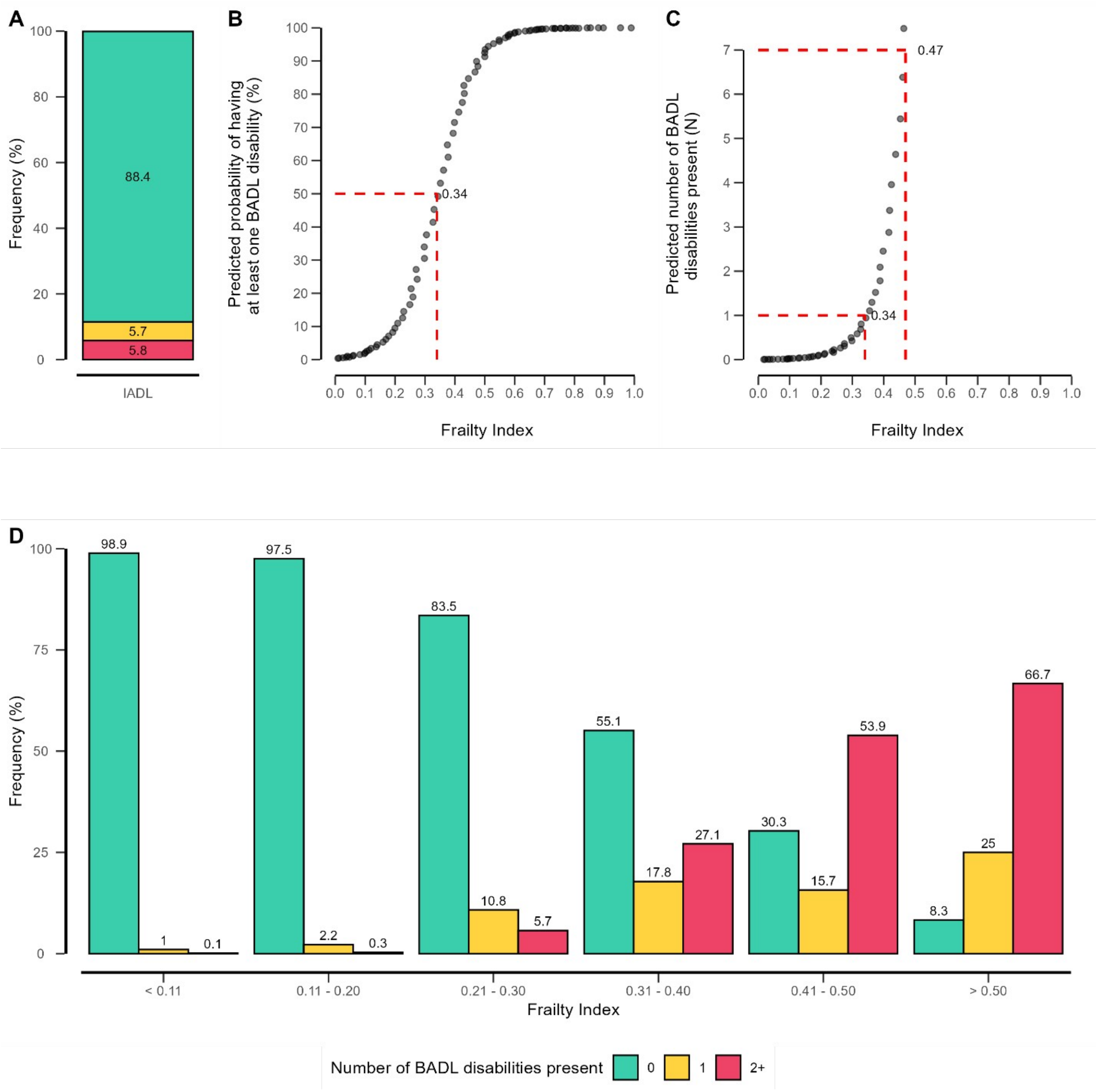

A
Frequency (%)
88.4
5.7
5.8
IADL
B
Predicted probability of having at least one BADL disability (%)
0.34
Frailty Index
C
Predicted number of BADL disabilities present (N)
0.47
0.34
Frailty Index
D
Frequency (%)
98.9
1
0.1
97.5
2.2
0.3
83.5
10.8
5.7
55.1
17.8
27.1
30.3
15.7
53.9
8.3
25
66.7
< 0.11
0.11 - 0.20
0.21 - 0.30
0.31 - 0.40
0.41 - 0.50
> 0.50
Frailty Index
Number of BADL disabilities present
0
1
2+

To examine the relationship between FI and BADL disability, FI was established without taking IADL and BALD-related items into account. Regressing the count of BADL disabilities to frailty level, adjusted for age, sex, race, marital status, and education.

**Parameter**	**IRR (95% CI) ***	**p**
Frailty Index		
< 0.11	Reference	-
0.11 - 0.20	2.52 (1.74 – 3.73)	<0.001
0.21 - 0.30	23.89 (17.24 – 34.07)	<0.001
0.31 - 0.40	92.94 (66.73 – 133.10)	<0.001
0.41 - 0.50	202.67 (138.30 – 304.37)	<0.001
> 0.50	191.68 (76.06 – 608.07)	<0.001

* IRR, Incidence Rate Ratio; 95% CI, 95% Confidence Interval.

Self-assessed health status

The presented plot displays the frequency of various self-assessed health statuses. Meanwhile, the accompanying table showcases the outcomes of a logistic regression analysis that links self-reported health to frailty level, accounting for age, sex, race, marital status, education, and disability (BALD/IALD).

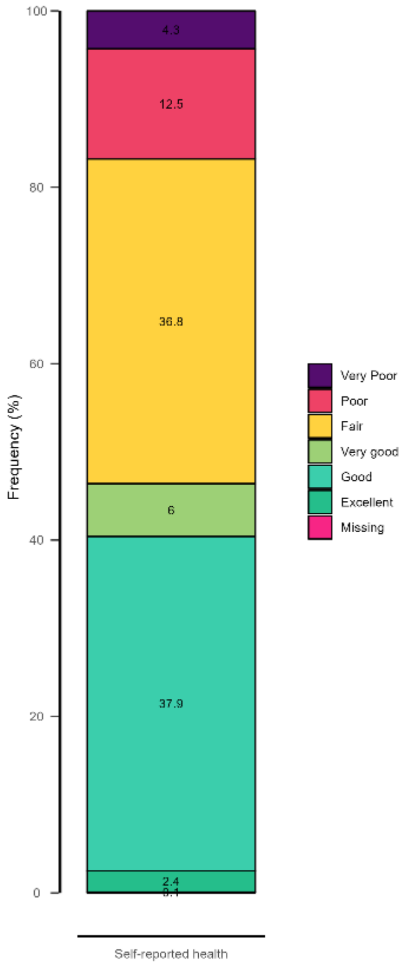

Parameter	OR (95% CI) *	p
Frailty Index		
< 0.11	Reference	-
0.11 - 0.20	3.55 (3.18 – 3.96)	<0.001
0.21 - 0.30	10.26 (8.86 – 11.88)	<0.001
> 0.30	24.12 (19.21 – 30.29)	<0.001

* OR, Odds Ratio; 95% CI, 95% Confidence Interval.

Healthcare use: doctor visits

Figure A presents prevalence estimates for doctor visits in the last 12 months. Meanwhile, Figure B depicts the number of doctor visits in the last 12 months. Figure C displays the frailty index values equivalent to the number of doctor visits in the last 12 months, adjusted for age, sex, race, marital status, and education (Negative Binomial model). Lastly, prevalence estimates for the number of doctor visits in the last 12 months based on frailty levels are provided in Figure D.

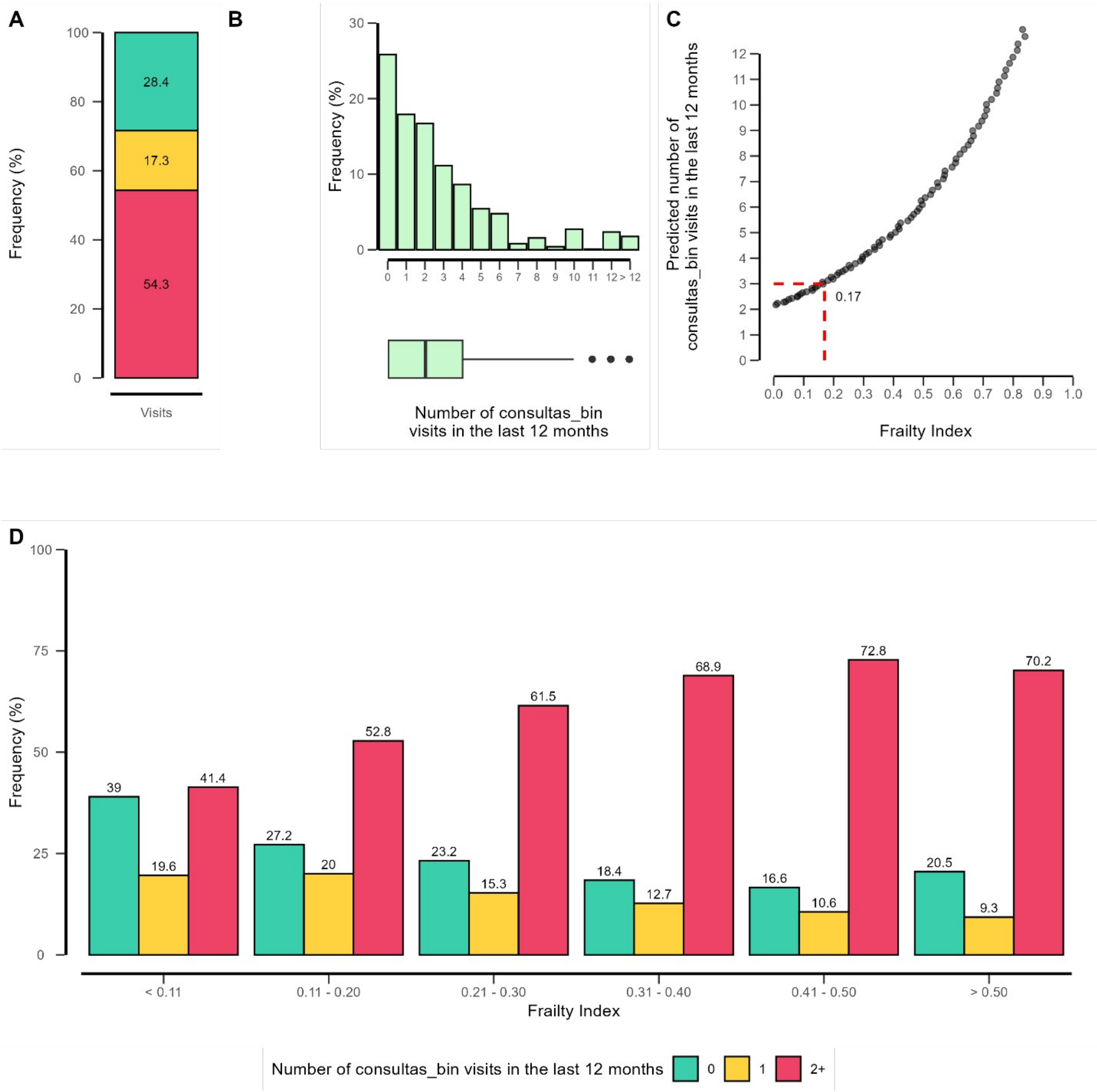

Healthcare use: specialist visits

To conduct this analysis, we narrowed the data only to include individuals who confirmed having visited a doctor at least once within the past 12 months. Figure A presents prevalence estimates for specialist visits in the last 12 months. Meanwhile, Figure B depicts the number of specialist visits in the last 12 months. Figure C displays the frailty index values equivalent to the number of specialist visits in the last 12 months, adjusted for age, sex, race, marital status, and education (Negative Binomial model). Lastly, prevalence estimates for the number of specialist visits in the last 12 months based on frailty levels are provided in Figure D.

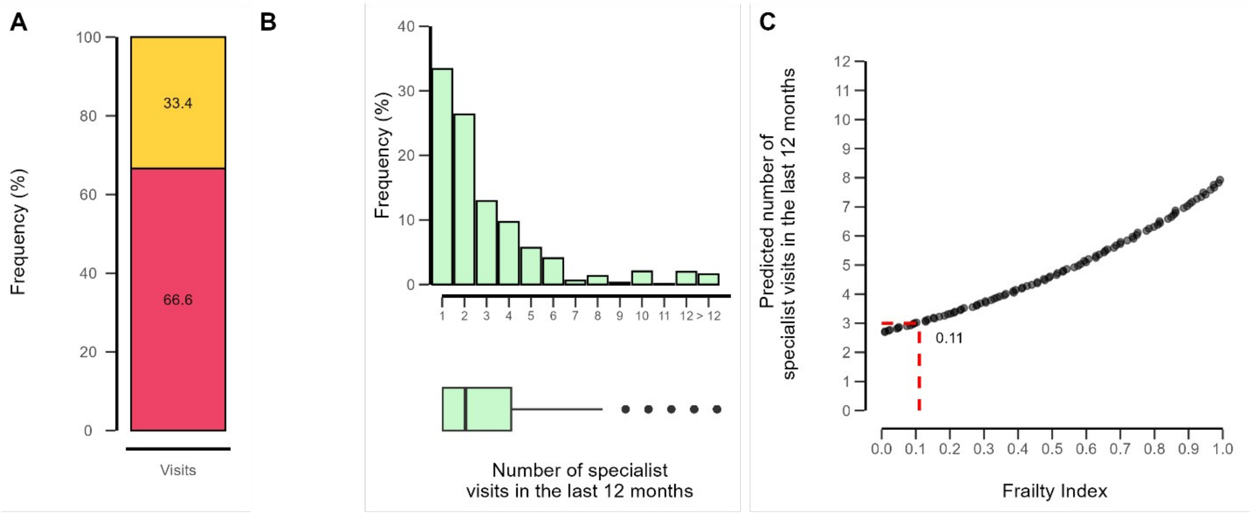

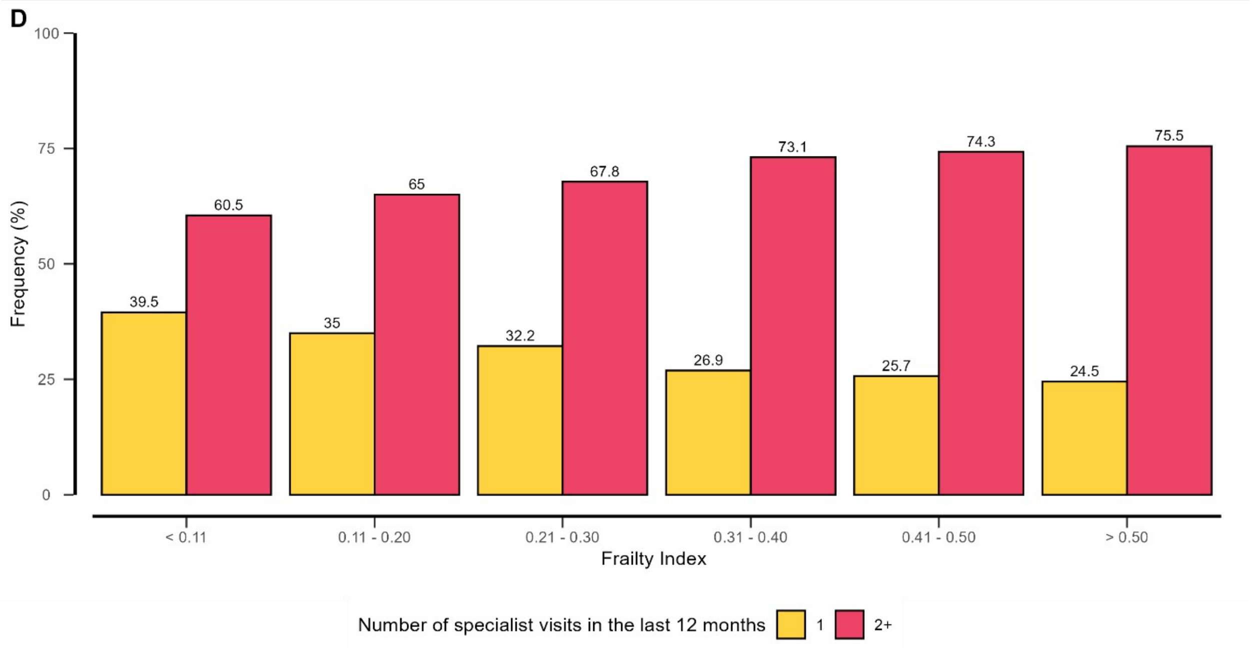

Healthcare use: hospitalizations

The estimated frequency of hospitalizations that occurred within the past 12 months can be observed in Figure A, whereas Figure B depicts the range of frailty index values equivalent to the number of hospitalizations in the last 12 months, adjusted for age, sex, race, marital status, and education (Negative Binomial model). Figure C illustrates the estimated frequency of hospitalizations within the same period, categorized according to frailty levels.

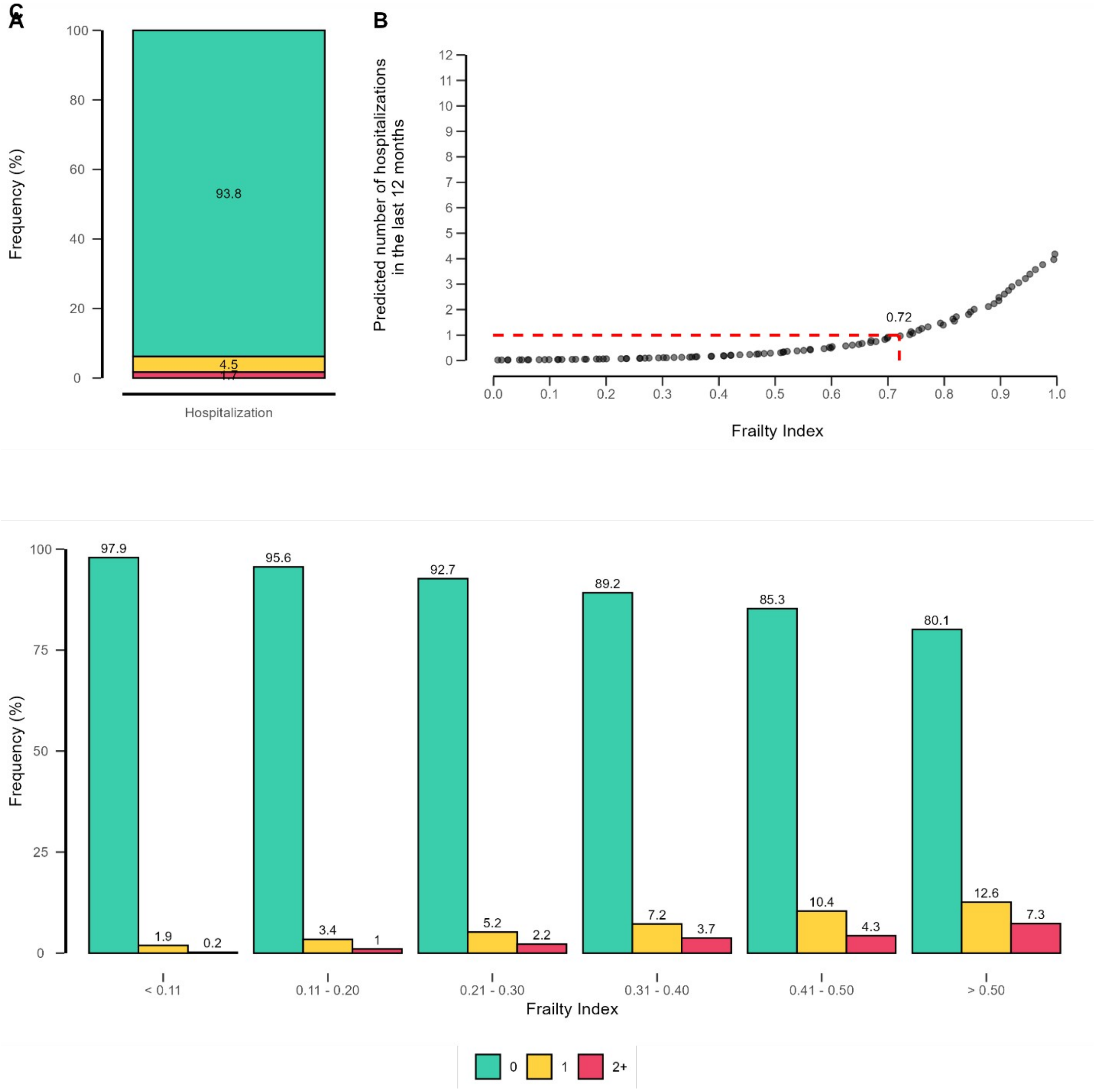

Frailty and healthcare use

Regressing the number of times the participant used healthcare services (e.g., doctor visits, specialists, hospitalizations) in the last 12 months to their frailty, adjusting for age, sex, and disability (ALD/IALD).

**Parameter**	**Incidence Rate Ratio (95% Confidence Interval)**		
	**Doctor visits**	**Specialists**	**Hospitalizations**
Frailty Index			
< 0.11	Reference	Reference	Reference
0.11 - 0.20	1.31 (1.23 – 1.39)	1.39 (1.27 – 1.52)	2.20 (1.61 – 3.01)
0.21 - 0.30	1.70 (1.58 – 1.83)	1.90 (1.72 – 2.11)	4.91 (3.54 – 6.81)
> 0.30	2.27 (2.07 – 2.49)	2.75 (2.41 – 3.14)	7.02 (4.77 – 10.34)
